# Simulation based virtual learning environment in medical genetics counseling: an example of bridging the gap between theory and practice in medical education

**DOI:** 10.1186/s12909-016-0620-6

**Published:** 2016-03-25

**Authors:** Guido Makransky, Mads T. Bonde, Julie S. G. Wulff, Jakob Wandall, Michelle Hood, Peter A. Creed, Iben Bache, Asli Silahtaroglu, Anne Nørremølle

**Affiliations:** Department of Psychology, University of Southern Denmark, Odense, Denmark; Faculty of Health and Medical Sciences, University of Copenhagen, Copenhagen, Denmark; Department of Education, University of Aarhus, Aarhus, Denmark; School of Applied Psychology and Menzies Health Institute Queensland, Griffith University, Gold Coast, Australia; Department of Cellular and Molecular Medicine, University of Copenhagen, Copenhagen, Denmark; Department of Clinical Genetics, Rigshospitalet, Copenhagen, Denmark

**Keywords:** Learning simulations, Virtual laboratory, Medical genetics education, Cytogenetics, E-Learning

## Abstract

**Background:**

Simulation based learning environments are designed to improve the quality of medical education by allowing students to interact with patients, diagnostic laboratory procedures, and patient data in a virtual environment. However, few studies have evaluated whether simulation based learning environments increase students’ knowledge, intrinsic motivation, and self-efficacy, and help them generalize from laboratory analyses to clinical practice and health decision-making.

**Methods:**

An entire class of 300 University of Copenhagen first-year undergraduate students, most with a major in medicine, received a 2-h training session in a simulation based learning environment. The main outcomes were pre- to post- changes in knowledge, intrinsic motivation, and self-efficacy, together with post-intervention evaluation of the effect of the simulation on student understanding of everyday clinical practice were demonstrated.

**Results:**

Knowledge (Cohen’s *d* = 0.73), intrinsic motivation (*d* = 0.24), and self-efficacy (*d* = 0.46) significantly increased from the pre- to post-test. Low knowledge students showed the greatest increases in knowledge (*d* = 3.35) and self-efficacy (*d* = 0.61), but a non-significant increase in intrinsic motivation (*d* = 0.22). The medium and high knowledge students showed significant increases in knowledge (*d* = 1.45 and 0.36, respectively), motivation (*d* = 0.22 and 0.31), and self-efficacy (*d* = 0.36 and 0.52, respectively). Additionally, 90 % of students reported a greater understanding of medical genetics, 82 % thought that medical genetics was more interesting, 93 % indicated that they were more interested and motivated, and had gained confidence by having experienced working on a case story that resembled the real working situation of a doctor, and 78 % indicated that they would feel more confident counseling a patient after the simulation.

**Conclusions:**

The simulation based learning environment increased students’ learning, intrinsic motivation, and self-efficacy (although the strength of these effects differed depending on their pre-test knowledge), and increased the perceived relevance of medical educational activities. The results suggest that simulations can help future generations of doctors transfer new understanding of disease mechanisms gained in virtual laboratory settings into everyday clinical practice.

**Electronic supplementary material:**

The online version of this article (doi:10.1186/s12909-016-0620-6) contains supplementary material, which is available to authorized users.

## Background

Fundamental knowledge in basic medical sciences such as molecular biology, cell biology, and genetics and proficiency in medical laboratory methods are core competencies for practicing physicians. With recent advances in diagnostic methods (e.g., genome analysis), it is also increasingly important that physicians have a strong scientific education. However, many medical students find it difficult to relate the foundational scientific education to the clinical practice of medicine, just as many struggle with learning how to counsel patients [1]. As early as 2003, the Institute of Medicine’s Clinical Research Roundtable identified an adequately trained workforce as a key challenge for better medical treatments [2].

The growing significance of translational medical science means that today’s medical educators face an increasing challenge of bridging the gap between theory and practice [3]. New laboratory techniques are often inaccessible to students because of prohibitory costs, time constraints, and safety concerns [4, 5]. This restricts medical education to providing a mainly theoretical understanding of these techniques and their application, rather than being able to leverage the advantages for real-world learning offered by new technologies [6]. This can leave students ill-prepared for the ongoing adaptations required by the continuously evolving field of translational medicine. Restricting training to theoretical knowledge can leave students finding much of what they are being taught in basic science curricula irrelevant to their future practice [3], undermining their engagement and motivation for learning [7].

In a recent position paper, Kurpinski et al. [8] (p. 1) concluded that “*specialized graduate education that teaches skills needed to negotiate the entire translational medicine continuum is invaluable for training the next generation of scientists, physicians, and managers”.* In addition, medical programs need to ensure that future doctors have high level interpersonal and communication skills so that they can engage fully with patients and their relatives. Developing these skills (e.g., patient interviewing and counseling skills) is difficult in large classroom settings. Additionally, making space in the curriculum for such training is challenging, especially as it must compete for time with high priority courses that provide training in core medical knowledge and techniques [1, 6]. Resolving the dilemma of providing adequate breadth and depth of a range of scientific and interpersonal skills requires novel ways of delivering training. One developing methodology that provides opportunities to do this is use of a simulation based learning environment, which is the focus of this paper.

### Simulation based learning environments in medical education

Physical simulations, such as training emergency care workers using mannequins, have been used widely for many years [9]. The emergence of digital simulation technologies now provides innovative ways for conveying medical knowledge by using case stories in highly realistic clinical scenarios. These simulations enable learners to see and interact with representations of natural phenomena that would otherwise be impossible to observe [10]. Additionally, the Gordon Commission’s [11] report on the Future of Assessment in Education concluded that simulations also have the potential to combine learning and assessment, which is essential for optimal advancement in the field of education.

Meta-analyses have found that deliberate practice through simulations is an efficient way to acquire medical and clinical expertise [12, 13]. Gamified laboratory simulations significantly enhanced students’ learning outcomes and motivation [14]. Medical students value highly the opportunity provided by simulations to systematically apply their theoretical knowledge to solve real-world problems in a safe and realistic setting [5]. For example, Issenberg and colleagues [15] demonstrated the benefits of simulation technology over traditional lectures in improving specific surgical technical skills, cardiovascular examination skills, and acquisition and retention of knowledge. Apart from developing technical skills, simulations are also effective in enhancing interpersonal and communication skills in health care professionals [16]. Thus, simulation technology offers advantages over traditional approaches in skills development and can be expected to improve engagement and motivation for learning.

Simulation technology is relatively inexpensive and safe, and allows students to work on complex problems and use time consuming techniques for analyzing patient samples – all in a virtual world. Examples of simulations for medical education include EdHeads (virtual knee replacement surgery) [17], Surgery squad (interactive, first-hand virtual surgery experience) [18], and Clinispace (an immersive, 3-D virtual hospital environment) [19]. As these examples illustrate, simulations are used widely to teach clinical practices, such as emergency care and surgery, but they are used less frequently to teach basic medical science [20]. One example is a Western Blotting simulation that was developed for medical education. The conclusions in this study were that simulation based learning environments have great potential for improving students’ development of diagnostic skills, but that further studies are required to determine the impact of laboratory simulations on student learning [21].

### Simulations to increase competencies and bridge the gap between theory and practice in medical education: medical genetics as an example

Medical genetics is one field in which medical education programs are struggling to keep pace with scientific and technological advances. This field has undergone rapid development, fueled by technological advances in molecular biology, including genetic testing methods and the growing body of knowledge on the genetic causes of both monogenic and complex inherited disorders. For example, the number of disorders for which genetic testing is available has increased 10-fold during the last 10 years [22]. In addition, genetics has entered the genomic era, where a growing understanding of the effects of genetic variation on common diseases is challenging our conception of the field [23].

A case-based approach to medical genetics using simulation technology has the potential to greatly enhance students’ understanding of underlying concepts and significantly increase their motivation [24]. The use of virtual patients in medical education has been shown to lead to increases in knowledge and clinical reasoning [25].

We propose that simulation technology will allow students to interactively choose and perform laboratory tests that will enhance their knowledge and understanding of the available genetic testing methods. Furthermore, we expect that the interpretation of the results through a case story will lead to an increase in students’ ability to draw valid conclusions relevant to genetic counseling.

**Fig. 1:**
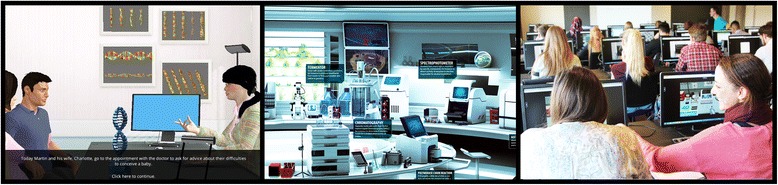
Screenshots of the virtual patient consulting session on the left, the virtual laboratory environment in the middle, and a picture of students using the simulation based learning environment on the right (permission to use the picture was obtained from all of the students who are included)

In collaboration with the simulation-focused company Labster, we developed a computer program with a case-based laboratory simulation for learning core concepts and skills in medical genetics and genetic. In this simulation, students are introduced to a young pregnant couple, where the fetus may suffer from a syndrome caused by a chromosomal abnormality. The students are able to make a genome wide analysis of the fetal DNA and karyotype in the virtual laboratory, and practice communicating their conclusions to the couple using a simulated genetic counseling approach (see Fig. 1 and Additional file 1 an overview of the simulation).

In such simulations, it is important that the case story is perceived as relevant [26], meaning that the genetic testing technologies introduced to the students need to be up to date. In our simulation, students perform amniocentesis and array comparative genome hybridization, grow cells, prepare chromosomes, and assess the karyotype. To ensure that the simulation is lifelike, reflecting state-of-the-art chromosomal analysis, we worked closely with medical doctors throughout the design phase.

The objectives of this study were to assess whether the learning simulation could increase students’: 1) understanding of the translation from molecular analysis to diagnosis and health decision-making in everyday clinical practice (i.e., bridge the gap between theory and practice in medical education); 2) knowledge of medical genetics; 3) intrinsic motivation for the topic of medical genetics; and 4) self-efficacy for performing medical genetics activities.

## Method

### Study sample

The sample comprised an entire class of 300 first-year undergraduate students (60 % female; 2 did not report gender) with either a major in medicine (84 %) or molecular biomedicine (16 %) from the University of Copenhagen in Denmark in the fall 2014 semester. Data were collected during the Medical Genetics course. Prior to undertaking the learning simulation, all students had completed a traditional 1-h lecture on subjects relevant to the simulation. All students completed the simulation evaluation and knowledge questions because these were a mandatory part of the curriculum; however, only 54 and 40 % of the sample had complete and valid responses to the self-efficacy and intrinsic motivation scales, respectively, as these were optional and included within a larger survey. An analysis of the difference between the group that completed and did not complete these optional scales is presented in the results section. Students were informed that the data would be used for research, and care was taken not to expose the students to any risk or burden (Helsinki Declaration article 17). Responses were anonymous (Helsinki Declaration article 24) and no stipend was provided. The study protocol was submitted to the Regional Committees on Health Research Ethics for Southern Denmark, who indicated that no written consent was required by Danish law.

### Training exposure and main outcome measures

The simulation session consisted of a 30-min pre-test to determine students’ baseline knowledge, intrinsic motivation, and self-efficacy; a 2-h session for the medical genetics simulation; and a 30-min post-test to evaluate the simulation and reassess students’ knowledge. Immediately following the post-test, students were given a survey that re-assessed their intrinsic motivation and self-efficacy along with other questions related to learning strategy and personality that were not part of this study.

Four questions were used to assess the student’s simulation experience in relation to its impact on their understanding of the translation from diagnosis into everyday clinical practice and health decision-making (e.g., “It is motivating to learn the concepts of Medical Genetics through a case story that resembles the real working situation of a doctor”). Responses were made on a 4-point Likert scale (1 = *Strongly disagree*, 4 = *Strongly agree*). Knowledge was assessed with 18 multiple choice questions (see Additional file 2 for a list of questions). Motivation was assessed using five questions adapted from the Interest/Enjoyment Scale from the Intrinsic Motivation Inventory [27] (see Additional file 2 for a list of questions). Self-efficacy for learning medical genetics was assessed using five questions adapted from the Motivated Strategies for Learning Questionnaire [28] (see Additional file 2 for a list of questions). Students indicated their responses to the motivation and self-efficacy items using a 5-point Likert scale (1 = *Strongly disagree*, 5 = *Strongly agree*). Evidence of validity and reliability was obtained for all scales, and post-hoc analyses showed that the items had good psychometric properties and acceptable fit to the Rasch measurement model [29].

## Results

### Translation to clinical practice

**Fig. 2:**
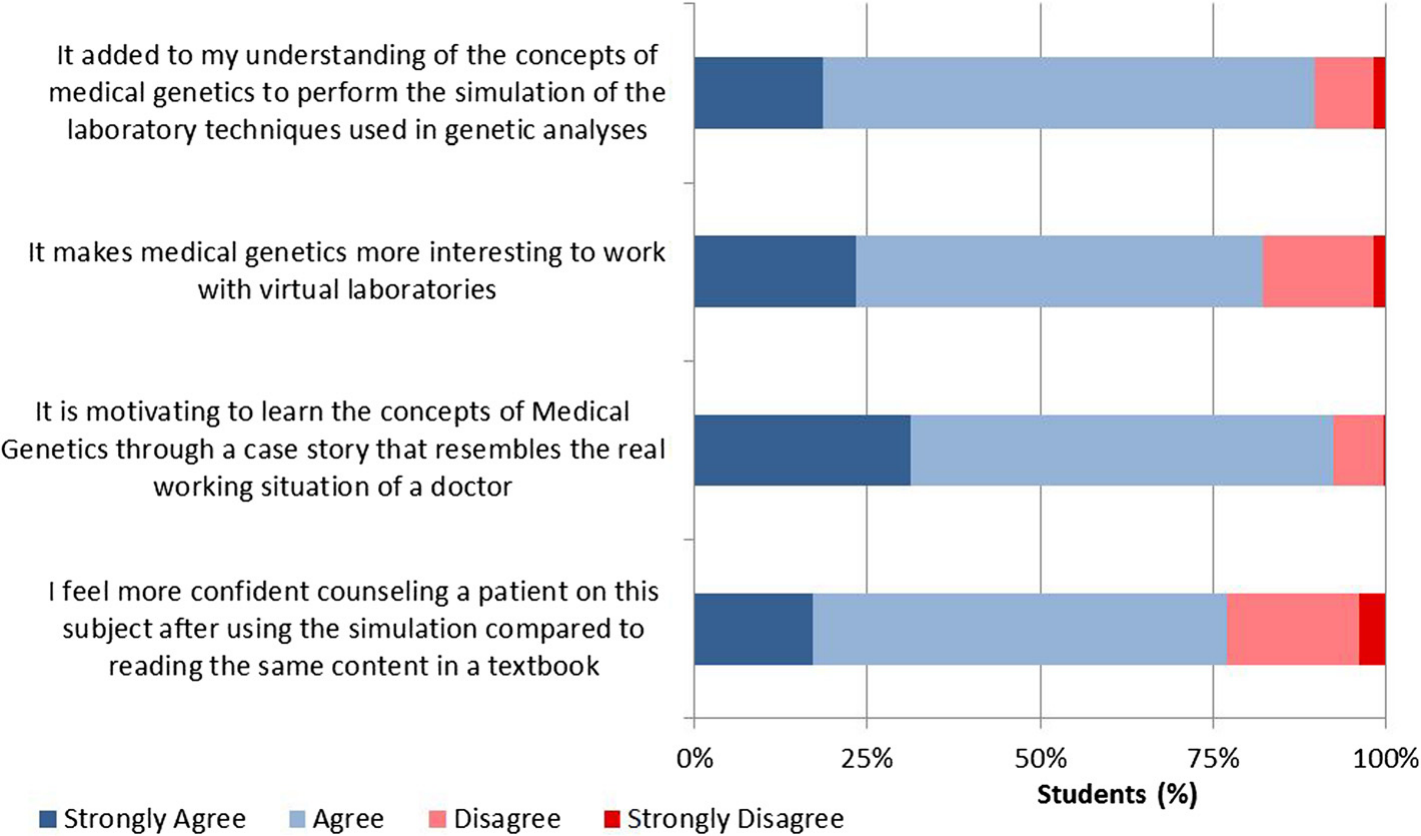
Illustration of student evaluations of the simulations impact on their understanding of the translation from diagnosis into everyday clinical practice and health decision-making

Students’ appraisals of the simulation based on the four evaluation questions indicated an increased understanding of the translation from diagnosis into everyday clinical practice and health decision-making (Fig. 2). Approximately 90 % of the students indicated that they agreed or strongly agreed that the performance of laboratory techniques added to their understanding of medical genetics, and 82 % indicated that medical genetics was more interesting because of working in a virtual laboratory. Furthermore, 93 % indicated that they were more motivated and gained confidence by having to work on a case story that resembled the real working situation of a doctor. Finally, 78 % indicated that they would feel more confident counseling a patient after the simulation.

### Knowledge gain

**Fig. 3:**
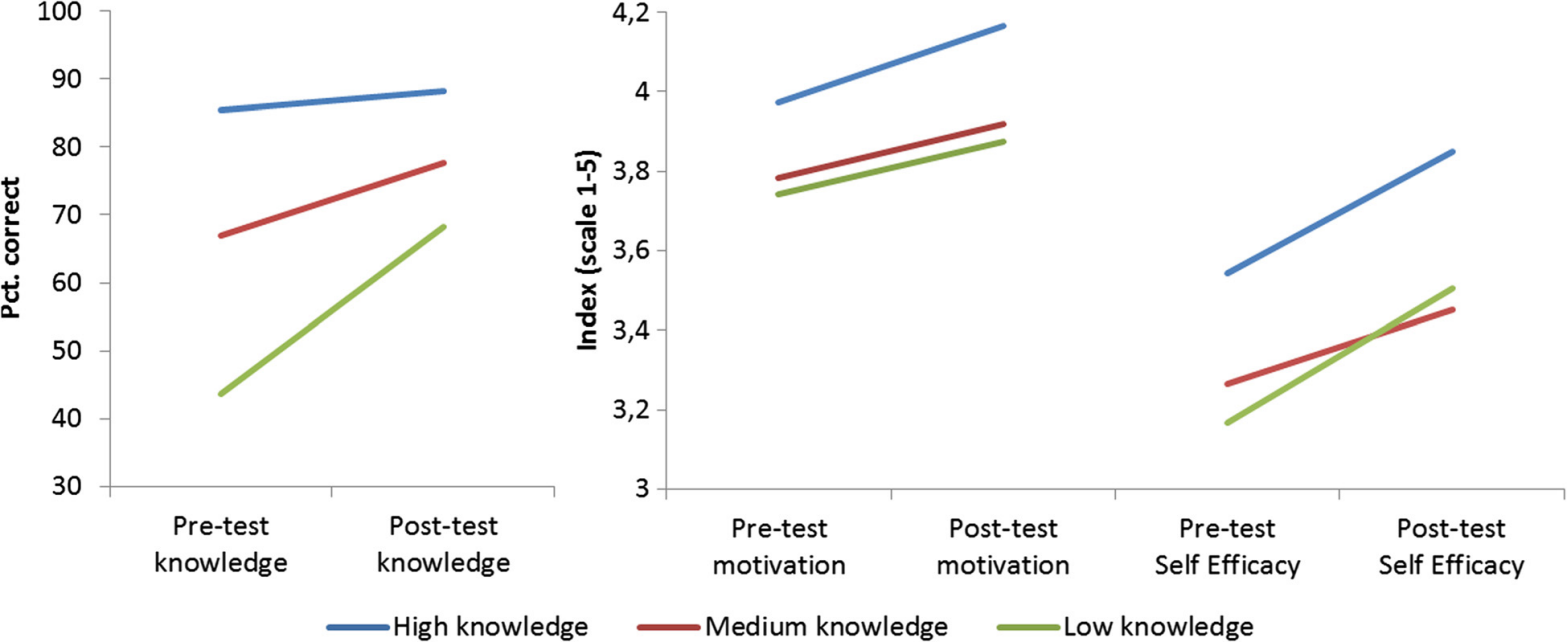
Mean (% correct) pre/post-test knowledge scores on the left, mean pre/post-test intrinsic motivation scores in the middle, and mean pre/post-test self-efficacy scores on the right for students with high, medium, and low knowledge at the pre-test

Paired samples *t*-tests showed a significant increase from a mean of 67 % correct on the pre-test to 79 % correct on the post-test, *t*_(299)_ = −14.07, *p* < .001; *d* = 0.73. This is a medium effect size, indicating an increase of 0.73 standard deviation units in knowledge. To determine if this gain differed depending on the students’ initial knowledge, the sample was divided into low (*N* = 86; pre-test score between 0 and 10), medium (*N* = 101; pre-test score between 11 and 13), and high (*N* = 113; pre-test score between 14 and 18) knowledge groups based on their pre-test score. The left-hand panel of Fig. 3 illustrates these results for knowledge. In general, there was a significant increase in knowledge as a result of the simulation for each knowledge group. The impact of the simulation was greatest for the low knowledge group, which increased their average score from 44 to 68 % correct, a significant increase of 24 %, *t*_(85)_ = 14.42, *p* < .001; *d* = 3.35. The medium knowledge group significantly increased their knowledge by 11 %, *t*_(100)_ = 10.87, *p* < .001; *d* = 1.45. Finally, the high knowledge group showed an average increase of 3 %, *t*_(112)_ = 3.69, *p* < .001; *d* = 0.36.

### Intrinsic motivation

Independent samples *t*-tests (using a Bonferroni corrected alpha of .008 to account for inflated Type I error rate) indicated that the group that completed the intrinsic motivation scale (40 % of the sample) did not significantly differ from the group that did not on pre-test knowledge (*p* = .711), post-test knowledge (*p* = .866), or on the four evaluation questions (*p*s from .071 to .522.

Students’ average intrinsic motivation for the topic of medical genetics increased significantly from a mean of 3.86 at pre-test (possible range = 1 to 5) to 4.02 at post-test, *t*_(118)_ = 4.23, *p* < .001; *d* = 0.24. The middle panel of Fig. 3 shows that intrinsic motivation was highest for the high knowledge group prior to the laboratory simulation, and the simulation had a significant positive effect on this group’s intrinsic motivation, *t*_(49)_ = 3.49, *p* = .001; *d* = 0.31. The medium knowledge group also showed a significant increase, *t*_(34)_ = 2.53, *p* = .016; *d* = 0.22. While scores for the low knowledge group increased, this change was not significant, *t*_(33)_ = −1.51, *p* = .141; *d* = 0.22.

### Self-efficacy

Independent samples *t*-tests (Bonferroni corrected alpha = .008) indicated that the group who completed the self-efficacy scale (56 % of the sample) did not differ significantly from the group that did not complete this on pre-test knowledge (*p* = .105), post-test knowledge (*p* = .012), or the four evaluation questions (*p*s from = .476 to .593).

Students’ average self-efficacy about their skills to perform medical genetics activities increased significantly from a pre-test mean of 3.36 (possible range = 1 to 5) to 3.62 at post-test, *t*_(161)_ = 8.46, *p* < .001; *d* = 0.46. The right-hand panel of Fig. 3 shows that prior to the simulation self-efficacy was highest for the high knowledge group, followed by the medium and low knowledge groups, respectively. Although all groups had significant increases in self-efficacy from the pre- to the post-test, the greatest effects were observed for the low knowledge, *t*_(37)_ = 4.56, *p* < .001; *d* = 0.61, and the high knowledge groups, *t*_(66)_ = 6.29, *p* < .001; *d* = 0.52. The self-efficacy of the medium knowledge group also increased significantly, *t*_(56)_ = 3.71, *p* < .001; *d* = 0.36; however, the effect size was weaker.

## Discussion

The results of this study indicate that a simulation based learning environment can increase the perceived relevance and interest of educational activities in medical education. This is very relevant for the future of translational medicine because an adequately trained workforce is crucial for the implementation of better medical treatments [2]. Ensuring an adequately trained workforce begins with medical educational programs at universities; however, it has proven to be a challenge to tie theoretical knowledge and rapid technological advances to practical settings, which can result in a gap between students’ perceptions of what they are being taught and the skills they are expected to have in their future careers as practitioners [3].

An important finding from this study was that students increased their confidence in their ability to counsel a virtual patient. This finding is in line with the results of a meta-analysis by Cook and colleagues who found positive effects for the use of virtual patients compared to both non-computer aided instruction and no intervention [25]. The current simulation adds to these existing ones with virtual patients as it affords not only the ability to interact with virtual patients, but also the ability to conduct virtual laboratory procedures. The current finding that nearly all of the students agreed that the performance of laboratory techniques added to their understanding of medical genetics attests to this added benefit. Further, the majority of students indicated that they subsequently found medical genetics more interesting because of the experience of working in the virtual laboratory. Consistent with the previous studies using virtual patients, we also found that most students indicated that they would feel more confident undertaking genetic counseling as a result of their interactions with the virtual patient. Thus, our results indicated that a combination of the virtual patient counseling interactions and simulated performance of actual laboratory procedures is ideal. As a result, nearly all students reported that they were motivated and more confident due to work on a case story that resembles the real working situation of a doctor. The findings suggest that laboratory simulations are valuable to train skills and competencies that are very hard to attain in traditional medical education, especially at the undergraduate level due to the difficulty of accessing real patients and the opportunity to work in real laboratories. These results support our expectations that simulations can increase student understanding of the translation from laboratory result analysis and diagnosis into everyday clinical practice and health decision-making.

The positive effects on students’ knowledge, intrinsic motivation, and self-efficacy were consistent with the students’ positive perceptions of the impact of the medical genetics simulation. The strength of these effects differed for high, medium, and low knowledge students. The high knowledge group did not show as big a gain in knowledge from the simulation, although this likely reflects a ceiling effect as they already had an average of 85 % correct at pre-test. This group also had the highest levels of intrinsic motivation and self-efficacy at both times. However, despite already being highest on motivation and self-efficacy, this group showed significant medium to large positive effects in motivation and self-efficacy as a result of the simulation. This can be anticipated to have long term, positive educational and career consequences resulting from the students’ application of their knowledge in clinically relevant settings and an increase in their perceived ability to counsel patients.

The low knowledge group showed the largest knowledge gain as a result of the simulation. This indicates that the simulation is particularly useful in promoting knowledge in weaker students. Furthermore, this group had a similar improvement in their self-efficacy to that seen in the high knowledge group. This could result in decreased drop-out rates and positive long term results as self-efficacy has been shown to be an important predictor for educational and career success [30–32]. These results are particularly enlightening because not all students benefit from traditional classroom environments: weaker students, for example, often report feeling bored, and subsequently demotivated, by not being able to meet high academic demands [33–35], and are considered to benefit from a more hands-on, problem-focused approach to learning [36–38].

### Recommendations for practice: simulation technologies as an integral part of future medical education?

Many medical schools are currently redesigning their curriculum to cater for the new developments and possibilities presented by modern technology. The results of this study provide evidence that redesigning medical education curricula with simulations is very promising because students valued working within a virtual laboratory context with case stories that resembled the real working situation of a doctor, and benefitted in their knowledge, motivation, and self-efficacy. This indicates multiple benefits because these outcomes have the potential to lead to positive long-term consequences for developing future doctors that go beyond the specific knowledge that is obtained in a single learning session. Intrinsic motivation is an important element in catalyzing long-term learning as well as engagement. High self-efficacy is important because there is evidence that belief in one’s ability to succeed can lead to greater educational and life outcomes than ones actual ability [31]. This is because self-efficacious students participate more readily, work harder, persist longer, and choose more challenging tasks and goals. Students’ beliefs about their efficacy to manage academic task demands can also influence them emotionally by decreasing their stress, anxiety, and depression [31].

A challenge for medical education is that the future generation of doctors needs to be able to transfer new understanding of disease mechanisms gained in the laboratory to new methods for diagnosis, therapy, and prevention, as well as the ability to translate these results into everyday clinical practice. If medical education programs are to meet this challenge, it is important to expose medical students to realistic laboratory and clinical scenarios as early as possible. We are beginning to see the impact of simulations in medical education and medicine in general, and the future could include fully simulated interactive clinic, hospital, and medical laboratories, which would enable students to experience an entire medical curriculum in an interactive environment. This will free teacher time and resources to focus on one-to-one interactions and training. Additional advantages are that simulations could be made available for a broad variety of students, including regional and remote students, which would mean that high quality medical education would no longer be limited to those able to attend specialized campuses; rather, state of the art medical education could be gained by anyone who is motivated to learn.

#### Limitations and future research

One of the limitations in this study is that there was no control group to compare the results of the virtual learning environment with other teaching methods. Although it is always an advantage to have a control group, the objective of this study was not to compare virtual learning environments to other teaching methods as this research question has been investigated previously [14, 21]. Rather than a control group, we assessed the outcome measures pre- and post- the simulation; therefore, effectively using participants as their own controls. In this study, we went beyond simply investigating cognitive outcomes such as increased knowledge and skills, by also examining non-cognitive outcomes; namely, intrinsic motivation and self-efficacy. This approach is in line with increasing evidence that cognitive as well as non-cognitive skills should be included as educational outcomes as both have significant short and long effects on educational [39].

Another potential limitation was that the same questions were used in the pre- and post-tests to assess knowledge. This could introduce potential bias because an increase in the score for the knowledge questions could be due to repeating the questions, rather than the simulation. The influence was limited by making sure that the students did not have access to the correct responses. Furthermore, the pre-test took place immediately before starting the simulation. Future research could consider using two different sets of items that are equated in the pre- and post-test to avoid this possible bias.

Another limitation was that we did not require participants to complete the post-test motivation and self-efficacy scales as these were part of a larger survey. As a result, there was a relatively large percentage of students who did not have complete and valid responses to these measures. The fact that completion of these measures was voluntary could introduce bias. Although a comparison of the groups who did and did not complete these measures showed no significant differences, future research should ensure that all participants complete all scales. This is particularly important given our findings in regard to these non-cognitive outcomes.

Future research is also needed to investigate how virtual simulation environments increase cognitive and non-cognitive outcomes in students as there is still limited research on this topic. There are two directions for this type of research. The first is to investigate causal relationships between psychological and educational constructs to facilitate a better understanding of the process of learning through simulation based tools. A second line of research is to investigate how different design components of simulation based learning environments affect cognitive and non-cognitive outcomes and whether individual differences variables such as personality, learning style, and computer or gaming experience play a role in preferences for specific design features.

## Conclusion

The simulation based learning environment increased students’ learning, intrinsic motivation, and self-efficacy, although the strength of these effects differed depending on their pre-test knowledge. In addition, the simulation based learning environment increased the perceived relevance of the medical educational activities to their future practice. Thus, the results provide evidence that simulations can help future generations of doctors transfer new understanding of disease mechanisms gained in virtual laboratory settings into everyday clinical practice.

## Ethics approval and consent to participate

Students were informed that the data would be used for research, and care was taken not to expose the students to any risk or burden (Helsinki Declaration article 17). Responses were anonymous (Helsinki Declaration article 24) and no stipend was provided. The study protocol was submitted to the Regional Committees on Health Research Ethics for Southern Denmark, who indicated that no written consent was required by Danish law.

## Consent for publication

Consent was obtained from all of the students who were included in the images in this manuscript.

## Availability of data and materials

All data is available through the first author of the manuscript.

## Additional files

Additional file 1: Video of the Simulation based Virtual Learning Environment used in this study. (MOV 12097 kb) Additional file 2: Questionnaire items used in this study. (DOCX 20 kb)
