# Supplementary material for: Simulation based virtual learning environment in medical genetics counseling: an example of bridging the gap between theory and practice in medical education
Source: BMC Med Educ. 2016 Mar 25;16:98. doi: 10.1186/s12909-016-0620-6 (PMC4807545; doi:10.1186/s12909-016-0620-6)
Supplement: Additional file 2: — Questionnaire items used in this study. (DOCX 20 kb) [file 12909_2016_620_MOESM2_ESM.docx]

**Additional file 2 questions used in the study.**

| **Intrinsic Motivation: 5 items** |
| --- |
| I enjoy working with Medical Genetics |
| Cytogenetic activities are fun to perform |
| Medical Genetics is boring |
| Medical Genetics does not hold my attention at all |
| I would describe Medical Genetics as very interesting |
| **Self-efficacy: 5 items** |
| I am confident and can understand the basic concepts of Medical Genetics |
| I am confident that I understand the most complex concepts related to Medical Genetics |
| I am confident that I can do an excellent job on the assignments and tests in the medical genetics exercises |
| I expect to do well in Medical Genetics |
| I am certain that I can master the skills being taught in Medical Genetics |

| **Knowledge: 18 items** | | | | |
| --- | --- | --- | --- | --- |
| Question | Response options (response in red is the correct answer) | | | |
| A young man is diagnosed with Klinefelter syndrome. What are his chances for having healthy children without medical assistance? | Men with Klinefelter syndrome are fertile. They do not have a significant increased risk of having abnormal offspring. | In most cases, men with Klinefelter syndrome are infertile | Men with Klinefelter syndrome are fertile, but they have a significantly increased risk of having abnormal offspring | In rare cases, men with Klinefelter syndrome are infertile. When they do have kids, their children will most likely carry translocations. |
| A woman experiences a spontaneous abortion. The aborted fetus has the karyotype 47,XY,+8. An investigation of a polymorphic DNA marker on chromosome 8 showed that the fetus and parents had the following alleles: Mother: allele 2 and 6, Father: allele 3 and 5, Fetus: allele 3 and 6. The trisomy can be explained by non-disjunction in meiosis. Which parent and in what meiotic division did this non-disjunction take place? | Only in the second meiotic division in either the mother or the father | Only in the mother in her first or second meiotic division | Only in the mother, and only in her second meiotic division | Only in the father in his first or second meiotic division |
| Which of the following karyotypes is most likely found in a three month old child of a parent who carries a balanced Robertsonian translocation 45,XX,der(14;21)(q10;q10)? | 46,XX,der(14;21)(q10;q10),+21 | 46,XX,der(14;21)(q10;q10),+14 | 47,XX,+21 | 45,XX,der(14;14)(q10;q10) |
| Individuals with three copies of autosomal chromosomes normally do not survive (except trisomies of e.g. 13, 18 and 21), whereas individuals with an extra X chromosome have relatively mild phenotypes. Why? | The X chromosome carries fewer genes than the autosomes | The X chromosome only determines the sex of the individual | All of the autosomes are acrocentric | The extra X chromosomes will be inactivated |
| Can a person with Turners syndrome become pregnant and give birth to a child? Select the most correct statement. | In very rare cases, she can become pregnant and give birth | Yes, she can become pregnant and give birth | It is most likely that she can become pregnant and give birth | She can become pregnant and give birth, but all of the children will have serious chromosomal abnormalities |
| Karyotype analysis of an infertile man reveals 46,XX. How can such a karyotype originate? | By duplication of the Y chromosome, so that it can no longer be recognized | By non-disjunction of the Y chromosome during the second meiotic division | By translocation of a part of the Y chromosome on to one of the X chromsomes | By deletion of the centromere region of the Y chromosome, which leads to loss of the Y |
| The figure shows the results of a fluorescent in situ hybridization (FISH) analysis. Which probes were used and what does the analysis reveal? | It used probes recognizing the centromere regions of chromosome 2 and 13 revealing a Robertsonian translocation between these two chromosomes | It used probes recognizing two frequent sequences in the genome showing the localization of these sequences in a normal man | It used paint probes (spectral karyotyping) recognizing chromosome 2 and 13 revealing an inversion on each of these two chromosomes | It used paint probes (spectral karyotyping) recognizing chromosome 2 and 13 to reveal a reciprocal translocation between these two chromosomes |
| A healthy man carries a balanced reciprocal translocation which can be described by: 46,XY,t(1;18)(p22;q23). What does this karyotype mean for his chances of getting normal and healthy children? | He can not have healthy children, as he will always passes on one or both of the two derivative chromosomes so that the child becomes a carrier of an unbalanced translocation | He can in very rare cases have healthy children, but only if he passes on the normal chromosomes 1 and 18 | He will only have healthy children since his translocation is balanced | He can have healthy children, if he passes on the normal chromosomes 1 and 18, or if he passes on the two derivative chromosomes so that the child becomes a carrier of the same balanced translocation |
| Which one of the following karyotypes do you NOT expect to find in a patient with Down's syndrome? | 46,XY,der(14;21)(q10;q10),+21 | 46,XX,der(21;21)(q10;q10) | 45,XX,der(21;21)(q10;q10) | 47,XX,+21 |
| If an infertile man has one Barr body in his cell nuclei, which of the following is most likely to be his karyotype? | 45,X | 47,XXY | 46,XY | 45,XY,der(22;22)(q10;q10) |
| When preparing the chromosomes for karyotyping, why do we add colcemid (or colchicine) to the cells? | To stop the cell division and reduce the number of cells in the final sample | To stimulate the cells to divide | To stop the cell division in metaphase where the chromosomes are most condensed | To label the chromosomes for visualization |
| What chromosomal rearrangement must have taken place in order to change the chromosome on the left into the derivative chromosome on the right? | Paracentric inversion | Pericentric inversion | Reciprocal translocation | Duplication |
| The person with the above karyotype has no symptoms of any genetic disease, but what is the risk for his children to have consequences due to the translocation he carries? | He can not have children | There is no risk of chromosome abnormalities for his children due to his translocation | All his children will have Down's syndrome | There is a risk for him to have children with Down's syndrome or miscarriages as well as possibility to have normal children |
| A child is born with the karyotype 47,XXX. This can be caused by non-disjunction, but what can we determine about in who and at which meiotic division? | It has to be in the mother, her first or second meiotic division | It can be in the mother, her first or second meiotic division, or in the second meiotic division in the father | It can only be in the father, his first meiotic division | It can only be in the second meiotic division, in either the mother or the father |
| A phenotypically normal boy has 45 chromosomes. His sister, who has Downs syndrome, has 46 chromosomes. Why? | The sister has a balanced Robertsonian translocation involving chromosome 21 | The boy only has one sex chromosome, while the sister has two | The boy has an unbalanced Robertsonian translocation involving chromosome 21 | Both children have a Robertsonian translocation involving chromosome 21. His is balanced, while hers is unbalanced with an extra chromosome 21 |
| A young couple has for two years tried to have children without success. The wife has experienced several miscarriages. Their karyotypes are 46,XX and 45,XY,der(22;22)(q10;q10). What different phenotypes can we expect in their future children? | We expect all children to have the same balanced translocation as the father, and hence be normal | We expect that they will have children with the same balanced translocation as the father and normal phenotype; all other possible karyotypes will lead to miscarriage | We expect that the couple is infertile, experiencing numerous miscarriages due to abnormal karyotypes | We expect a high risk of children with Down's syndrome, and a high frequency of micarriages |
| Array CGH analysis of a young child with developmental abnormalities revealed that the short arm of chromosome 4 was present in only one copy. What does this mean? | The child most likely suffers from Wolf-Hirschhorn syndrome due to a deletion on 4p | The child suffers from Turner syndrome due to a deletion on 4q | The child most likely suffers from Cri du Chat syndrome | The child has Edwards syndrome due to a duplication on 4p |
| A person with the karyotype 46,XX,der(18)t(12;18)(p13;q12) carries 46 chromosomes. A part of the short arm of chromosome 12 has replaced a part of the long arm of chromosome 18 in one of the chromosome 18 pair (the derivative chromosome 18) due to a translocation, inherited from her father. What does this mean? | She has only one copy of the short arm of chromosome 18 | She has three copies of the long arm of chromosome 18 | She has three copies of the short arm of chromosome 12 | She has only one copy of the long arm of chromosome 12 |
